# Pleural Fibroma; A meandering path to surgical removal

**DOI:** 10.12669/pjms.311.5517

**Published:** 2015

**Authors:** Shafqat Hassan, Syed Shirjeel Husain, Muhammad Amim Anwar, Saema Saeed

**Affiliations:** 1 Dr. Shafqat Hassan, Cardio-Thoracic Surgeon, Memon Medical Institute Hospital, Karachi, Pakistan.; 2 Dr. Syed Shirjeel Husain, ICU In-charge, Memon Medical Institute Hospital, Karachi, Pakistan.; 3 Dr. Muhammad Amim Anwar, In-charge Department of Anesthesia, Memon Medical Institute Hospital, Karachi, Pakistan.; 4 Dr. Saema Saeed, Medical officer ICU, Memon Medical Institute Hospital, Karachi, Pakistan.

**Keywords:** Solitary fibrous tumor, Lung mass, Counseling

## Abstract

A 52 Year old male was admitted with respiratory distress. Radiological examination revealed a large mass in patient’s right hemi thorax with mediastinal shift and partial lung collapse. Biopsies previously done conferred the diagnosis of solitary fibrous tumor; however, in order to avoid a surgical resection, patient didn’t follow the adviced procedure. After thorough counseling, surgical resection was done with few post operative complications and patient recovered well with ability to perform his daily activities with partial support. The histopathology results showed solitary fibrous tumor. Apart from pneumonia and local wound infection, patient status was well for the next six week follow-up.

## INTRODUCTION

Localized pleural fibromas are a definite clinical entity. Their origin is much debated. Though most arise from the mesothelial cell, occasionally some arise from the pleural fibroblast.^1^

Over the years this entity has acquired a number of synonyms, including localized fibrous tumor, benign mesothelioma, localized fibrous mesothelioma, submesothelial fibroma, and pleural fibroma. The usage of term ‘mesothelioma’ for this tumor is discouraged because of potential confusion with diffuse malignant mesothelioma, a much more serious disease.

The term solitary fibrous tumor (SFT) was first mentioned in scientific literature by Wagner in 1870. However the first discussion of its clinical and pathological properties was explained by Klemperer et al. in 1831. Approximately 78-88% of the SFT’s are benign and 12 to 22% are malignant.^2^^,^^3^ Though they can arise virtually from any site in the soft tissue, pleural fibromas mostly originate in the visceral pleura upto 80%, while 20% arise from the parietal pleura.^4^

Symptoms of SFT’s are non-specific and mostly include chest symptoms like cough, dyspnea or chest pain which mostly correlates with tumor size. CT Scan and MRI remain the choice for determination of the relationship of mass with surrounding tissue in case of large masses. Malignant transformation is also not uncommon.

## CASE REPORT

A fifty two year old male was admitted via Emergency department with complaints of shortness of breath. Patient had history of intermittent episodes of shortness of breath for last two years with each episode being worsened from the previous one. The last episode started one month ago restricting his physical activity only to few steps; Shortness of breath is partially relieved by lying laterally. It was also associated with productive cough with white scanty, non-foul smelling sputum, occasionally mixed with blood. Patient also received ATT (anti tuberculosis therapy) for last fifteen days without any betterment in his status. He holds past two year record of admissions, where he was treated for pneumonia initially; later two admissions were done with extensive workup firstly in August 2012 and latter January 2013. Biopsies were done both the time and both confer the diagnosis of solitary fibrous tumor. Patient was advised for followup in both institutes, but never returned and finally landed in our institute in order to avoid a possible surgical resection.

Clinically patient was restless, tachycardic and tachypneic but maintained oxygen saturation on 3-4 liter of oxygen. His chest examination showed decrease right sided movements with dull percussion note, decrease breath sounds. Lab investigations further revealed anemia with Hemoglobin of 8.0 gm/dl, blood gases showed mild hypoxia with PO2 of 80mm Hg while rest of his metabolic panel & co-agulation profile were within normal limit.

**Fig.1:**
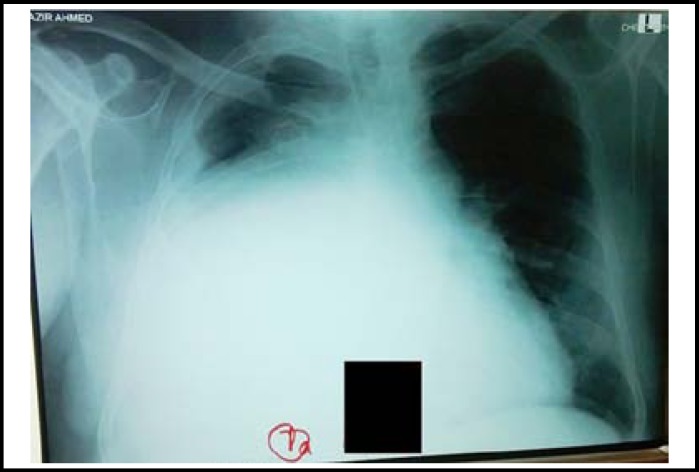
Chest X ray: showing a huge homogenous opacity on the right side displacing mediastinum to the left.

**Fig.2:**
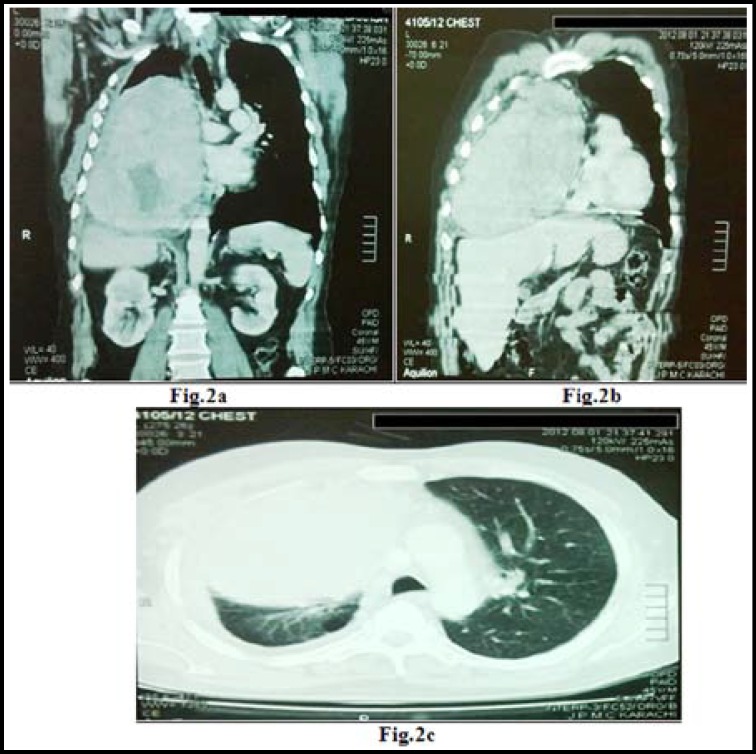
a, b, c: CT Scan of chest; shows a large heterogeneous mass with few sparks of calcification occupying almost whole of the right hemi thorax. Medially the mass is abutting and pushing superior vena cava, Aorta, right pulmonary artery and heart. Mass is also causing compression effects on the right hemi-diaphragm.

Chest X-ray showed a large homogenous opacity on right side with mediastinal shift. CT Scan of chest confirmed the finding of a large heterogeneous mass with few sparks of calcification occupying almost whole of the right hemi thorax. Medially the mass was abutting and pushing superior vena cava, Aorta, right pulmonary artery and heart. Mass was also causing compression effects on the right hemi-diaphragm. It is displacing the mediastinal contents on to the left and caused compression collapse of right medial and lover lobe. Minimal pleural effusion with pleural thickening was also noted. (Fig. 1 and 2)

**Fig.3:**
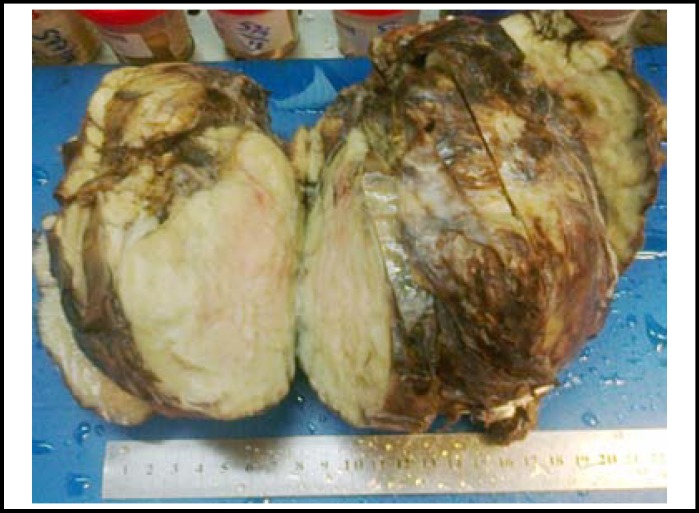
View of a single multi nodular encapsulated pale white firm piece of tissue after removal

After thorough discussion and multiple settings; patient was counselled for surgical removal of the mass and its catastrophic outcome in case of further delay. Explorative thoracotomy was done and a huge extra pleural mass was removed. Right upper lobe of the lung was also adherent to the mass. Careful incision was performed to remove the mass preserving the surrounding vascular structures with minimal blood loss. The size of excised mass 24 x 20cm x 8cm and it was 3150gm in weight. (Fig.3). The lower and middle lobe collapse was relieved partially and later fully. Two drains were placed and wound was approximated.

The pathological examination revealed a single multi nodular encpsulated pale white firm piece of tissue. On serial sectioning two collapsed cavities were also indentified with larger one measuring 5 x5 cm filled with yellow pleurant exudate. A flap of pleura id identified too measuring 12x6 cm.

Microscopic examination revealed demarcated lesion composed of spindloid cells. The nuclei are oval to elongated with evenly dispersed chromatin. Cytoplasm is abundant. Scattered mitotic figures are seen. Areas of hyalinization and fibrosis are noted. Conclude as neoplastic lesion; solitary fibrous tumor as possibility.

Patient was kept on mechanical ventilation, but, as per plan, the ICU team could not wean him off in next two days. Patient also developed fever spikes and later developed ventilator associated pneumonia. Tracheal cultures revealed acinetobacter. Intravenous anti-biotics were given as per sensitivity and after six days patient was finally extubated. Drains were removed on third day of surgery. Physical capacity continues to increase slowly and patient was able to walk short distances without any discomfort.

## DISCUSSION

Pleural fibrous tumor are rare tumors and due to their non-specific symptoms are often misdiagnosed as pleural effusions, pneumonia, Tuber-culosis or else.^5^ Consequently, the failure or initial management led to further investigation and diagnosis of the sinister wasting clinically important time period, when the in growing tumor is causing increased pressure effects. The difficulty can further be bundled in developing countries where clinicians solely clinical acumen for diagnosis or due to scarcely available diagnostic resources.^5^

Prognosis of solitary fibrous tumor is excellent and only 8% of the disease will reoccur after first resection, with the recurrence usually cured after additional surgery.^3^ However the rate of recurrence is higher in malignant SFT with poor prognosis, most of the patient dying within two year of diagnosis.^1^

If Malignant SFT is associated with pleural effusion then recurrence and mortality is recorded even higher.^6^ Complete resection yields better long term survival as compared to partial resection and decrease the incidence of recurrence too.^6^

CT scan is a valuable tool and contrast enhanced images help not only in diagnosis but also modify surgical decisions. MRI is also comparable in this regard.^7^ In our case, though the patient was earlier diagnosed on basis of Chest X ray and treated as Tuber-culosis, but sooner he was diagnosed on biopsy as solitary fibrous tumor. Yet, his surgical resection was delayed for at least seven month indicating lack of counselling; a pivotal role in modern day clinical practice and ethics.

Counselling plays a vital role in development of doctor-patient relationship, patient’s satisfaction and course of their disease.^8^ On the contrary, if our patient had been counselled properly, a neoplastic lesion could have been operatively removed earlier with lesser complications and morbidity.

In view of it, we emphasize the practicing clinicians especially in developing country like ours that counselling in comparison with therapeutics is equivalent and if not done properly may result in conversion of an amendable disease to latter more morbid state/disease.

## Authors Contribution:

**SH:** Critical revision for important intellectual content and final approval of the version to be published.

**SSH:** Concept, Design, Acquisition of Data and Critical revision for important intellectual content.

**MAA:** Concept, Design and Critical revision for important intellectual content.

**SS:** Design and Acquisition of Data.
